# The role of the jaw subdomain of peptidoglycan glycosyltransferases for lipid II polymerization

**DOI:** 10.1016/j.tcsw.2018.06.002

**Published:** 2018-06-20

**Authors:** Avinash S. Punekar, Firdaus Samsudin, Adrian J. Lloyd, Christopher G. Dowson, David J. Scott, Syma Khalid, David I. Roper

**Affiliations:** a School of Life Sciences, University of Warwick, Coventry CV4 7AL, United Kingdom; b School of Chemistry, University of Southampton, Southampton SO17 1BJ, United Kingdom; c School of Biosciences, University of Nottingham, Sutton Bonington Campus, Leicestershire LE12 5RD, United Kingdom; d ISIS Neutron and Muon Spallation Source and Research Complex at Harwell, Rutherford Appleton Laboratory, Oxfordshire, United Kingdom

**Keywords:** Bacterial cell wall, Lipid II, Peptidoglycan synthesis, Glycosyltransferases, Jaw subdomain, Antibiotic resistance, Moenomycin A, Antimicrobial peptide

## Abstract

Bacterial peptidoglycan glycosyltransferases (PGT) catalyse the essential polymerization of lipid II into linear glycan chains required for peptidoglycan biosynthesis. The PGT domain is composed of a large head subdomain and a smaller jaw subdomain and can be potently inhibited by the antibiotic moenomycin A (MoeA). We present an X-ray structure of the MoeA-bound *Staphylococcus aureus* monofunctional PGT enzyme, revealing electron density for a second MoeA bound to the jaw subdomain as well as the PGT donor site. Isothermal titration calorimetry confirms two drug-binding sites with markedly different affinities and positive cooperativity. Hydrophobic cluster analysis suggests that the membrane-interacting surface of the jaw subdomain has structural and physicochemical properties similar to amphipathic cationic α-helical antimicrobial peptides for lipid II recognition and binding. Furthermore, molecular dynamics simulations of the drug-free and -bound forms of the enzyme demonstrate the importance of the jaw subdomain movement for lipid II selection and polymerization process and provide molecular-level insights into the mechanism of peptidoglycan biosynthesis by PGTs.

## 1 Introduction

**Fig. 1:**
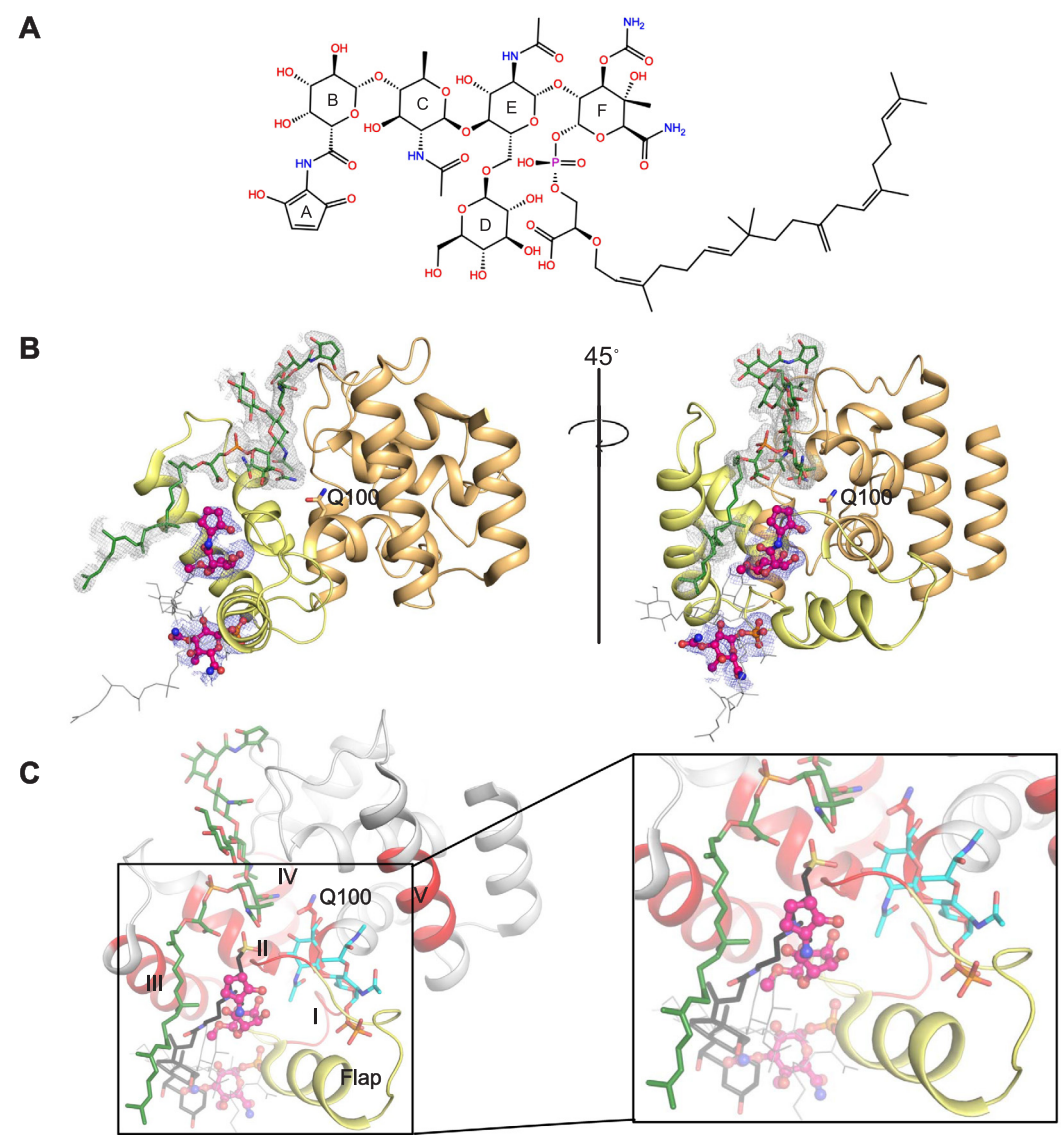
(A) Structure of moenomycin A (MoeA). (B) Overall view of the re-refined structure of SaMGT E100Q mutant with bound MoeA (PDB ID code 6FTB). The jaw subdomain is colored yellow and head subdomain is colored orange. ${\text{MoeA}}_{\mathrm{DON}}$ (green) is shown in stick representation and ${\text{MoeA}}_{\mathrm{JAW}}$ (pink) is shown in ball-and-stick representation. Polder electron density map (Liebschner et al., 2017) for ${\text{MoeA}}_{\mathrm{DON}}$ (grey mesh) and ${\text{MoeA}}_{\mathrm{JAW}}$ (blue mesh) is contoured at 2σ. Unmodelled parts of ${\text{MoeA}}_{\mathrm{JAW}}$ (due to no visible electron density) is shown in gray colored line representation. (C) SaMGT with bound lipid II analog (cyan colored shown in stick representation) in the acceptor site (PDB ID code 3VMT) and the PGT domain of *Aquifex aeolicus* PBP1A (PDB ID code 2OQO) with bound CHAPS (black colored shown in stick representation) are superimposed on 6FTB. Only 6FTB (gray colored cartoon) is shown for clarity. ${\text{MoeA}}_{\mathrm{DON}}$ and ${\text{MoeA}}_{\mathrm{JAW}}$ are shown as in B. PGT motifs I to V (colored red) and flap (colored yellow) is shown. (For interpretation of the references to colour in this figure caption, the reader is referred to the web version of this article.)

The bacterial cell wall is made of peptidoglycan (PG) — a mesh-like rigid structure that protects bacteria against the high osmotic pressure of the cytoplasm (Vollmer et al., 2008). PG is synthesized from its precursor lipid II (Glc*N*Ac-Mur*N*Ac(pentapeptide)-undecaprenyl pyrophosphate) by the concerted action of membrane-bound enzymes containing the PG glycosyltransferase (PGT) and transpeptidase (TP) domains (Sauvage et al., 2008, Lovering et al., 2012). The PGT module is found in two different families of PG synthases; as a single domain in monofunctional glycosyltransferases (MGTs) and as the N-terminal domain of the bifunctional class A penicillin-binding proteins (PBPs) (Sauvage et al., 2008). Multiple sequence alignment of the PGT domain show five conserved sequence motifs (I to V, Supplementary Fig. 1A). Crystal structures of several PGTs, both in the ligand-free form and in complex with a specific PGT inhibitor moenomycin A (MoeA, Fig. 1A) and/or lipid II analogue (Lovering et al., 2007, Yuan et al., 2007, Lovering et al., 2008, Yuan et al., 2008, Heaslet et al., 2009, Sung et al., 2009, Fuse et al., 2010, Han et al., 2011, Huang et al., 2012, King et al., 2017) confirm that these motifs contain residues important for lipid II binding and catalysis. This essential function by PGT makes it a potential antibiotic target. Therefore, a thorough understanding of the PGT mechanism would help in the rational design of novel PGT inhibitors that could be developed as antibiotics (Galley et al., 2014).

The cell walls of Gram-positive and Gram-negative bacteria differ in the types of phospholipids they contain, but in both cases, there is a net negative charge at the membrane surface at physiological pH (Teixeira et al., 2012, Malanovic and Lohner, 2016). Lipid II is also found in this phospholipid environment but is a low-abundant molecule (less than 1 percent of total membrane phospholipids) and mostly found in the regions of membrane close to cell division and elongation (Kramer et al., 2004, Breukink and de Kruijff, 2006). Therefore, it is important to understand at the molecular level, how PGT domains selectively capture lipid II molecules from a crowded milieu of membrane phospholipids.

The PGT domain adopts an all α-helical globular fold with an extended groove that divides this domain into two subdomains—a large head subdomain containing features similar to the bacteriophage λ-lysozyme (Lovering et al., 2007) and a small jaw subdomain containing features specific to the glycosyltransferase family 51 (GT_51_) (Supplementary Fig. 1B). The extended groove forms the PGT enzymatic cavity and the hydrophobic surface at the base of the jaw subdomain enables it to embed into the extracellular face of the cytoplasmic membrane and access the lipid II substrate (Lovering et al., 2007, Derouaux et al., 2013). Another prominent feature of the jaw subdomain is a protruding dynamic ‘flap’ region (Supplementary Fig. 1), which is highly disordered in most of the PGT structures, but resolved in the structures of *Staphylococcus aureus* monofunctional glycosyltransferase (SaMGT) (Heaslet et al., 2009, Huang et al., 2012), *S. aureus* penicillin-binding protein 2 (SaPBP2) (Lovering et al., 2008) and *Escherichia coli* penicillin-binding protein 1b (EcPBP1b) (King et al., 2017). The flap region adopts an α-helix-loop structure and serves as a steric barrier to physically separate the PGT enzymatic cavity into two substrate-binding sites—a donor site for the lipid-bound growing glycan chain and an acceptor site for the lipid II (King et al., 2017). The flap region has been proposed to play an active role in the processive catalytic mechanism of PGTs (Derouaux et al., 2013, Egan et al., 2015, Sauvage and Terrak, 2016). The schematic models describing the PGT catalytic mechanism suggest that the flap prevents dissociation of the elongating glycan chain and a localized unfolding or a structural rearrangement of flap regulates the translocation of the elongating glycan chain from the acceptor site to the donor site (Yuan et al., 2007, Lovering et al., 2008, Bury et al., 2015, King et al., 2017); however, the actual mechanism of the growing glycan strand translocation at the active site of PGT is not well understood.

In our exploration of the mechanism of PGT enzymes, we have investigated and re-refined the 2.1 Å crystal structure of MoeA-bound SaMGT E100Q mutant [Protein Data Bank (PDB) ID code  3HZS] originally solved by a research team at Pfizer (Heaslet et al., 2009). In addition to the MoeA bound in the donor site of SaMGT (henceforth referred to as ${\text{MoeA}}_{\mathrm{DON}}$) as seen in the original 3HZS structure, re-refinement has revealed a second molecule of MoeA bound to the jaw subdomain (henceforth referred to as ${\text{MoeA}}_{\mathrm{JAW}}$). Isothermal titration calorimetry (ITC) results revealed positive cooperativity in binding of two MoeA molecules to PGT. Further, we have used a 2D hydrophobic cluster analysis (HCA) method and molecular dynamics (MD) simulations to study the PGT domain and investigate whether any common structural and physicochemical features shared by the jaw subdomain play a functional role in the lipid II selection and polymerization process.

## 2 Material and methods

### 2.1 Cloning, expression and purification

SaMGT (residues Q28-R269) was cloned, expressed and purified as described previously (Huang et al., 2012). Further details in the Supplementary Information.

### 2.2 Isothermal titration calorimetry

Calorimetric titrations of SaMGT with MoeA were performed on a VP-ITC microcalorimeter (MicroCal) at 25 °C and measured in duplicate. The gel filtration purified SaMGT was concentrated and dialyzed overnight against buffer A (20 mM Tris-HCl, pH 8.0, 200 mM NaCl, 2 mM N-decyl-β-d-maltopyranoside (DM)) at 4 °C. All solutions were degassed by gentle stirring under vacuum for 15 min. The dialysis buffer A was used to prepare MoeA solution. The sample cell (1.445 mL) was filled with 20 μM SaMGT and the syringe (280 μL) with 450 μM of the titrating MoeA. Each ITC experiment consisted of a preliminary 2 μL injection followed by 27 successive 10 μL injections. Each injection lasted 20 s with an interval of 200 s between consecutive injections. The solution in the sample cell was stirred at 351 rpm throughout the experiment. The heat response data for the preliminary injection was discarded and the rest of the data was used to generate binding isotherm. The data were fit using the sequential binding site model included in the Origin 7.0 (MicroCal). Thermodynamic parameters, including association constant (Ka), enthalpy (ΔH), entropy (ΔS), and binding stoichiometry (N), were calculated by iterative curve fitting of the binding isotherm. The Gibb’s free energy (ΔG) was calculated using ΔG=ΔH-TΔS.

### 2.3 Model building and structure refinement

Re-refinement of MoeA-bound SaMGT E100Q mutant (PDB ID code  3HZS) was performed using the program BUSTER 2.10.2 (Bricogne et al., 2009) and model building was done in Coot (Emsley and Cowtan, 2004). Polder omit maps were calculated using the *phenix.polder* tool in the PHENIX software package (Liebschner et al., 2017). Geometrical restraints for the ligands were generated using the Grade Web Server (Smart and Womack, 2014) and Rhofit program (Smart et al., 2011) was used for automated fitting of ligands into the difference electron density.

### 2.4 Hydrophobic cluster analysis

The primary sequences of the PGT domain in MGTs and class A PBPs were converted into hydrophobic cluster analysis (HCA) plots (Gaboriaud et al., 1987) using the HCA plot server at Mobyle@RPBS (Callebaut et al., 1997). The HCA plot relies on the 2D helical representation of the amino acid sequence obtained after duplication of an unrolled cylinder in which the amino acid residues follow a classical α-helical pattern (3.6 residues per turn). The hydrophobic residues (F, V, L, I, M, Y, and W) are surrounded by a mosaic and few residues are specially displayed: P (star), G (diamond), T (square) and S (dotted square). Since the protein secondary structure is associated with the clusters of hydrophobic residues in the plot, this method allows distinguishing between the β-strands and α-helices (Lemesle-Varloot et al., 1990). Usually, alanine is not included as a part of the hydrophobic cluster; however, the presence of highly conserved alanine residues in few of the α-helices in PGT domain suggested us to include this residue, which has significantly helped in the analysis of the HCA plot. HCA similarity scores were obtained by calculating the number of hydrophobic amino acids using the following formula: HCA homology score (%) = [(2CR/RC1 + RC2) × 100], where CR is the number of hydrophobic residues which are in correspondence between the compared sequences and RC1 (RC2) is the number of hydrophobic residues in protein 1 (protein 2).

### 2.5 Molecular dynamics

The crystal structure of full-length membrane-bound SaMGT (PDB ID code  3VMT) was used to model the N-terminal transmembrane α-helix in the apo SaMGT structure and was embedded within a model of *S. aureus* cell membrane containing 57% phosphatidylglycerol (PG), 38% lysyl-phosphatidylglycerol (Lys-PG), and 5% diphosphatidylglycerol (DPG; also known as cardiolipin). The lipid composition of this model membrane was based on experimentally determined values (Gould and Lennarz, 1970, Haest et al., 1972) and its structure has been previously validated (Piggot et al., 2011). The N-terminal α-helical domain of SaMGT was inserted into the membrane using the g_membed protocol (Wolf et al., 2010). This system was solvated and 0.15 M NaCl was added. A short 1 ns equilibration simulation was performed whereby the heavy atoms in the protein were positionally restrained using a force constant of 1000 kJ mol^−1^. The temperature was kept at 310 K using the velocity rescale thermostat with a time constant of 0.1 ps (Bussi et al., 2007). The pressure was maintained at 1 atm using a semi-isotropic coupling to the Parrinello-Rahman barostat using a time constant of 1.0 ps (Parrinello and Rahman, 1981). The particle mesh Ewald method was used to calculate the electrostatic interactions, whereby a real-space cut-off of 0.9 nm was utilised (Essmann et al., 1995). The van der Waals interactions were truncated at 1.4 nm with long-range dispersion correction applied to the energy and pressure. All bonds were constrained using the LINCS algorithm to allow for a 2 fs time step (Hess et al., 1997). After the equilibration, the positional restraints on the heavy atoms were removed and three independent production runs, each for 500 ns, were performed at 310 K (Table S2). One simulation was performed at a higher temperature of 323 K for 750 ns to accelerate the dynamics. The MoeA molecule was parameterised using the Automatic Topology Builder (Koziara et al., 2014). The ${\text{MoeA}}_{\mathrm{DON}}$ bound SaMGT was then equilibrated inside the *S. aureus* membrane model using the same protocols described for the apo protein. Three independent 500 ns simulations were performed with this protein-ligand complex at 310 K. All simulations were performed using the GROMACS package version 4.6.7 (Van Der Spoel et al., 2005, Hess et al., 2008), the GROMOS 54A7 force field (Schmid et al., 2011), and the SPC water model (Berendsen et al., 1981). Built-in GROMACS analysis tools and VMD (Humphrey et al., 1996) were employed to analyse the trajectories.

## 3 Results

### 3.1 Re-refined structure of the MoeA-bound SaMGT E100Q mutant (PDB ID code 3HZS).

The authors of the high-resolution crystal structure of the MoeA-bound SaMGT E100Q mutant (PDB ID code  3HZS) (Heaslet et al., 2009) reported a tightly ordered phosphate ion bound in a pocket (formed by residues His107 to Leu112) at the base of the jaw subdomain that they proposed could represent the binding site for the pyrophosphate (PP) moiety of the acceptor lipid II substrate, however the authors did not use any phosphate containing salts in the SaMGT purification and/or crystallization experiments. This prompted us to re-examine 3HZS structure to resolve this apparent discrepancy. Our re-analysis has revealed several positive difference electron density features, which we manually inspected to interpret the electron density and identify appropriate ligands that could be correctly modelled into these features, resulting in an updated model (PDB ID code  6FTB) with improved overall statistics from the original as shown in Supplementary Table 1.

The difference map produced by the *phenix.polder* tool in PHENIX (Liebschner et al., 2017) shows for the first time a complete C25 moenocinol lipid tail of ${\text{MoeA}}_{\mathrm{DON}}$ modelled into the structure (Fig. 1B). The saccharide ring D of ${\text{MoeA}}_{\mathrm{DON}}$ has been marginally shifted to fit accurately into the electron density (Supplementary Fig. 2A) and this improved fitting subsequently revealed a bifurcated electron density extending from the C2 carbon atom of ring D suggesting that the functional group at this position is not OH, but an ‘unknown’ functional group that would make a bifurcated hydrogen bonds to the oxygen atoms of the phosphate moiety and saccharide ring F (Supplementary Fig. 2B). None of the MoeA derivatives currently known explain this potential adduct (Ostash and Walker, 2010). Additional electron density was observed in the groove accommodating ring A of ${\text{MoeA}}_{\mathrm{DON}}$ which is consistent with a non-ionic surfactant: glyceryl monolaurate (Supplementary Fig. 2C) used during cell lysis procedure (Heaslet et al., 2009). The polar end of glyceryl monolaurate makes strong hydrogen bond interactions with the side chains of Asp184, Tyr196 and the backbone atoms of Pro226, Ser227, Val228, Tyr229 and Asn230. These residues are part of a patch of conserved residues in the head subdomain of the GT surface, which is probably of functional significance (Supplementary Fig. 2C). Structural superimposition showed that glyceryl monolaurate binds in a different pocket than the CHAPS bound to the PGT domain of *Aquifex aeolicus* PBP1A (PDB ID code  2OQO) (Fig. 1C) (Yuan et al., 2007).

Our analyses also revealed a large positive electron density extending from the bound phosphate ion at the base of the jaw subdomain (His107 to Leu112), along the external surface, connecting to an additional area of positive electron density at the mouth of a deep pocket just below the catalytic site (Fig. 1B). This deep pocket is surrounded by the conserved residues in what we now define as motif I (Asp101, His107, Gly109, Asp111), motif II (Gln129, Gly130, Gly131, Ser132, Thr133, Gln136) and motif III (Lys153), suggesting its functional significance. The difference density from the polder map was sufficient to fit the A and B rings of MoeA at the mouth of the deep pocket and ring F connecting the bound phosphate ion at the base of the jaw subdomain (Fig. 1B). Saccharide rings C, D, E and the C25 moenocinol lipid tail could not be modelled due to a lack of significant difference density. The overall electron density suggested that sections of a second MoeA molecule (i.e. ${\text{MoeA}}_{\mathrm{JAW}}$) bound to the jaw subdomain could be modelled at this site. Strikingly, the tail of the CHAPS molecule in 2OQO occupies the same position in space as the A and B rings of ${\text{MoeA}}_{\mathrm{JAW}}$ and the steroidal ring of CHAPS occupies approximately the same space as rings C, D and E of ${\text{MoeA}}_{\mathrm{JAW}}$ (Fig. 1C). Superimposing the structure of lipid II analog-bound SaMGT (PDB ID code  3VMT) onto 6FTB showed that the A and B rings of ${\text{MoeA}}_{\mathrm{JAW}}$ snuggly fit in a pocket adjacent to the lipid II analog (bound in the acceptor site) and the motif II residues (Gly130 to Ser132) act like a steric barrier to separate this well-conserved pocket from the catalytic site (Fig. 1C). The ring B of ${\text{MoeA}}_{\mathrm{JAW}}$ forms hydrogen bonds with the side chains of Asp101, Gln136, the backbone carbonyl oxygen atom of Phe110 and the water molecules in the pocket.

No other changes were found in the protein chain overall except for extension of the map at the N-terminus by one residue so that Glu59 is now included. Superimposition of the original (PDB ID code  3HZS) and re-refined (PDB ID code  6FTB) SaMGT-MoeA structures yield a root-mean-square deviation (RMSD) of 0.092 Å over the ${\text{C}}_{\alpha }$ atoms of the 208 amino acid residues.

### 3.2 Positive cooperativity in binding of MoeA to PGT

**Fig. 2:**
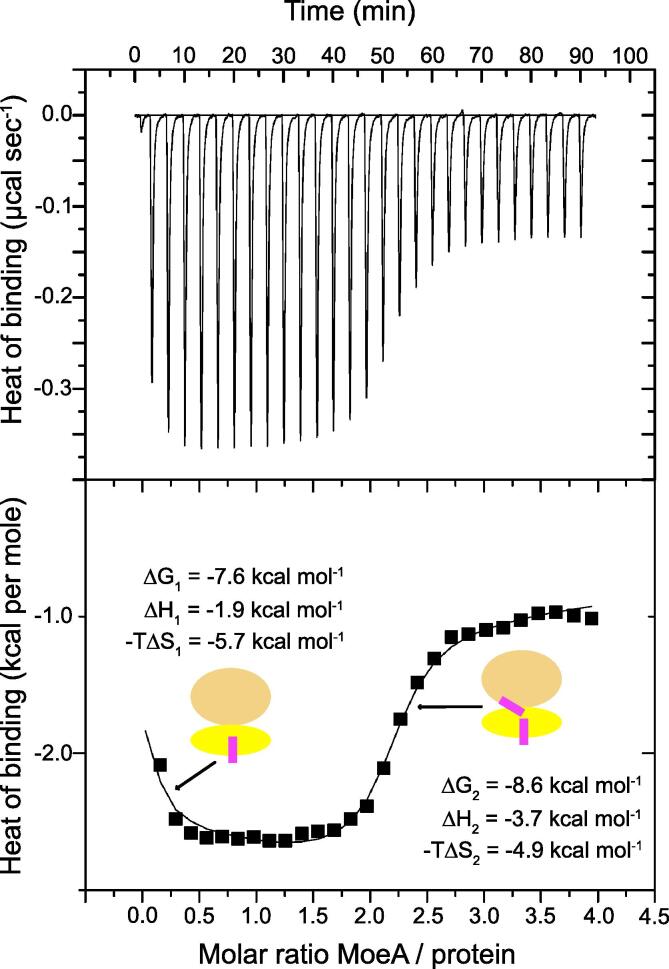
Energetics of cooperative sequential binding of MoeA to SaMGT. ITC heat response (top) and binding isotherm (bottom) of the calorimetric titration of MoeA into SaMGT. A cartoon representation of SaMGT (head subdomain colored orange and jaw subdomain colored yellow) and MoeA (pink) is shown in the bottom chart. The solid line in the bottom chart represents the fit to a sequential binding site model. The dissociation constant $\left({\text{K}}_{d}\right)$ for the ${\text{MoeA}}_{\mathrm{JAW}}$ and ${\text{MoeA}}_{\mathrm{DON}}$ binding steps are 2.8 μM and 0.54 μM, respectively. (For interpretation of the references to colour in this figure caption, the reader is referred to the web version of this article.)

Discovery of ${\text{MoeA}}_{\mathrm{JAW}}$ bound in the jaw subdomain of the 3HZS structure prompted us to characterize the binding of MoeA to wild-type full-length SaMGT. We used isothermal titration calorimetry (ITC) to measure the association free energy (ΔG) and its enthalpic (ΔH) and entropic components (ΔS) for MoeA binding to SaMGT. The biphasic nature of the binding isotherm (Fig. 2) clearly points to two non-equivalent sites in SaMGT with a significant difference in binding affinity for MoeA.

Macroscopic apparent binding affinities were determined to be ${\text{K}}_{\mathrm{app}1}$ of 3.56 · 10^5^ ± 2.7 · 10^4^ M^−1^ and ${\text{K}}_{\mathrm{app}2}$ of 1.87 · 10^6^ ± 1.1 · 10^5^ M^−1^. Due to statistical effects, it would be expected that ${\text{K}}_{\mathrm{app}1}=4\cdot {\text{K}}_{\mathrm{app}2}$. However, it can be seen that ${\text{K}}_{\mathrm{app}1}\approx \frac{1}{5}{\text{K}}_{\mathrm{app}2}$ indicating positive cooperativity. As noted by Velazquez-Campoy and co-workers (2015) we can calculate the intrinsic binding constant (*k*) and cooperativity factor (α) for a cooperative binding event from the apparent binding constants as:

$$K_{\mathrm{app}1}=2k$$

$$K_{\mathrm{app}1}K_{\mathrm{app}2}=\alpha k^{2}$$

giving values of *k* = 1.78 · 10^5^ ± 1.4 · 10^4^ M^−1^ and α=21.3. As α>4, this demonstrates positive cooperativity. The measured enthalpy change between the two binding steps, $\Delta {\text{H}}_{1}\text{-}\Delta {\text{H}}_{2}$, is +1.8 kcal mol^−1^. Thus, binding of ${\text{MoeA}}_{\mathrm{JAW}}$ is accompanied by a favourable enthalpy change.

### 3.3 Structural similarity between the PP-binding motifs of PGT jaw subdomain and antimicrobial peptides

**Fig. 3:**
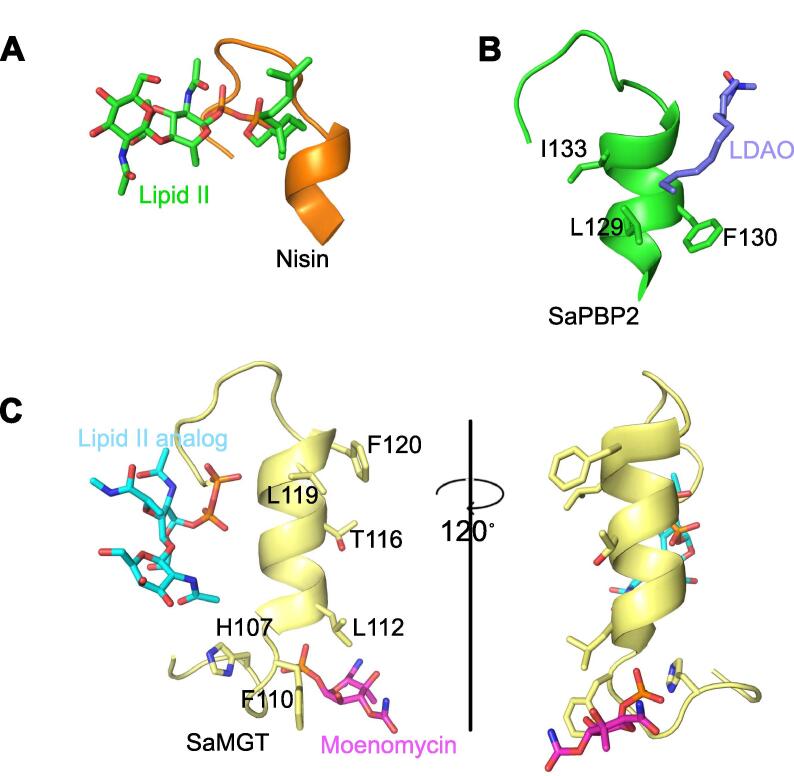
Structural similarity between the PP-binding motif in the cationic antimicrobial peptide nisin, *Staphylococcus aureus* penicillin-binding protein 2 (SaPBP2) and *S. aureus* monofunctional glycosyltransferase (SaMGT). (A) Solution structure of nisin-lipid II complex (PDB ID code 1WCO). The N-terminal part of nisin (residues 1–12 colored orange) encages the PP moiety of lipid II (colored green). (B) Flap segment of SaPBP2 (residues 126–141 in chain B of PDB ID code 3DWK colored green) interacts with the acyl tail of lauryldimethylamine N-oxide (LDAO, colored blue). (C) Flap segment of SaMGT (residues 104–128 in PDB ID code 6FTB colored pale yellow) with bound ${\text{MoeA}}_{\mathrm{JAW}}$ (colored pink) and modelled lipid II analog (colored cyan) by superpositioning with PDB ID code 3VMT. (For interpretation of the references to colour in this figure caption, the reader is referred to the web version of this article.)

The hydrogen bond interactions between the phosphate ion of the ${\text{MoeA}}_{\mathrm{JAW}}$ and the backbone atoms of the well-conserved motif I residues implies the presence of a common PP-binding structural motif in all PGT enzymes for lipid II recognition and binding (Supplementary Fig. 1 and see Discussion). To characterize the function of the PP-binding motifs in PGT enzymes, we used the Omokage shape similarity search ( https://pdbj.org/emnavi/omo-search.php) (Suzuki et al., 2015) to scan the PDB for proteins with overall fold similarity for PP binding. The first search for the segment covering motif I–flap region of SaMGT (residues Arg103-Thr116 in PDB ID code  3HZS) revealed the solution structures of cysteine deleted protegrin-1 (CDP-1, PDB ID code  2MQ2) (Mohanram and Bhattacharjya, 2014) and a nontoxic bacterial membrane anchor peptide (PDB ID code  1VM4) (Wang et al., 2005) as the best hits, suggesting its possible role in membrane perturbation and anchoring (see following section). Remarkably, the second search for the segment covering the “tip” region of the flap of SaMGT (residues Arg117-Val128 in PDB ID code  3HZS) revealed the solution structures of the cationic antimicrobial peptide (AMP) nisin-lipid II complex (PDB ID code  1WCO) (Hsu et al., 2004), mersacidin-lipid II complex (PDB ID code  1MQZ) (Hsu et al., 2003) and the X-ray crystal structure of racemic plectasin (PDB ID code  3E7R) (Mandal et al., 2009) as the top-most hits. For simplicity and clarity in understanding, we will restrict our comparison of the flap region of PGTs with the nisin-lipid II complex (Fig. 3).

The N-terminal part of nisin (residues 1–12) adopts a unique nest-like motif called “PP cage” to bind the PP moiety of lipid II by hydrogen bonds from the amide groups of the nisin backbone (Fig. 3A) (Hsu et al., 2004). Deleting the C-terminal tail of the peptide inhibits the pore-forming activity of nisin but still maintains its lipid II PP-binding ability (Chan et al., 1996). We found a striking similarity between the “PP cage” motif of nisin and the “tip” region of the flap of SaMGT (Fig. 3A and C) and equivalent residues in PGTs of SaPBP2 (Fig. 3B) and EcPBP1b. A key notable difference is that the “PP cage” motif follows N- to C- direction in nisin and C- to N- direction in PGTs. Superimposition of the PDB ID code  3VMT (lipid II analog bound in the acceptor site of SaMGT) and 3HZS show that the PP moiety of the lipid II analog comes very close to binding the PP cage-like motif at the “tip” of the flap (Fig. 3C).

### 3.4 Hydrophobic cluster analysis of the PGT domain

Multiple sequence alignment of the PGT domain in MGTs and Class A PBPs show conserved amino acids in the motifs I to V, whereas the rest of the protein sequences share low levels of sequence identity, especially in the flap segment (only one conserved alanine in the α-helix, Supplementary Fig. 1A). 3D-structures of SaMGT (PDB ID code  3HZS, Heaslet et al. (2009)), SaPBP2 (PDB ID code  3DWK (Lovering et al., 2008)) and EcPBP1b (PDB ID code  5HLB (King et al., 2017)) have shown that despite low sequence identity, the flap adopts an α-helix-loop structure. Simple 1D sequence comparison cannot be used to study the protein domains showing very limited amino acid relatedness. In such cases, 2D hydrophobic cluster analysis (HCA) method (Gaboriaud et al., 1987, Callebaut et al., 1997, Lemesle-Varloot et al., 1990) has successfully identified conserved regions including motifs of functional significance in β-glycosyltransferases (Saxena et al., 1995) and catalytic residues in glycosyl hydrolases (Henrissat, 1990). Here, we have used the HCA method to study the PGT domain in MGTs and class A PBPs.

**Fig. 4:**
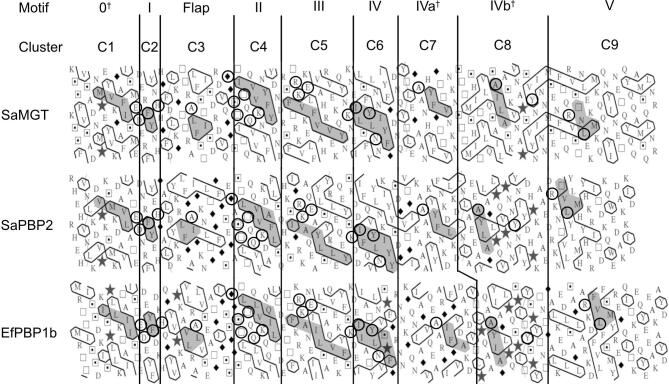
Aligned HCA plots of the PGT domain in monofunctional glycosyltransferases (MGTs) and class A penicillin-binding proteins (PBPs). Only three sequences are shown here due to space limitations. UniProt accession numbers: *Staphylococcus aureus* MGT (SaMGT, Q7A0I6), *S. aureus* PBP2 (SaPBP2, Q9R744) and *Enterococcus faecalis* PBP1b (EfPBP1b, I3U3N7). A comprehensive HCA plot with more aligned sequences is shown in Supplementary Fig. 3. The standard one-letter code for amino acids is used except for glycine (♦), proline (★), serine  and threonine (□) respectively. The vertical lines indicate proposed correspondence between the compared sequences. The strictly conserved residues (in the multiple sequence alignment Supplementary Fig. 1A) are indicated with a black circle. The conserved and conservatively substituted amino acids within the hydrophobic clusters (shaded in gray) suggest a structural and functional relationship. † indicates motifs identified in this study.

A 2D HCA plot showed that nine hydrophobic clusters (C1 to C9) cover the entire PGT domain (Fig. 4 and Supplementary Fig. 3). Clusters C1 and C6 to C9 are part of the head subdomain and clusters C2 to C5 are part of the jaw subdomain. In particular, our HCA analysis has identified three new motifs in the head subdomain that we are proposing for the first time – cluster C1 (motif 0), cluster C7 (motif IVa) and cluster C8 (motif IVb). Comparison of the HCA plots revealed a similarity in the shapes and patterns of the clusters made by the hydrophobic residues suggesting their structural and functional roles. The four highly conserved hydrophobic clusters C1, C2, C4 and C6 (HCA homology score >90%) make the core of the PGT domain. Clusters C3, C7, C8 and C9 are highly divergent in terms of their precise shape; nevertheless, arrangement of few hydrophobic residues is roughly the same, which gives a minimal pattern to these clusters (Fig. 4 and Supplementary Fig. 3).

**Fig. 5:**
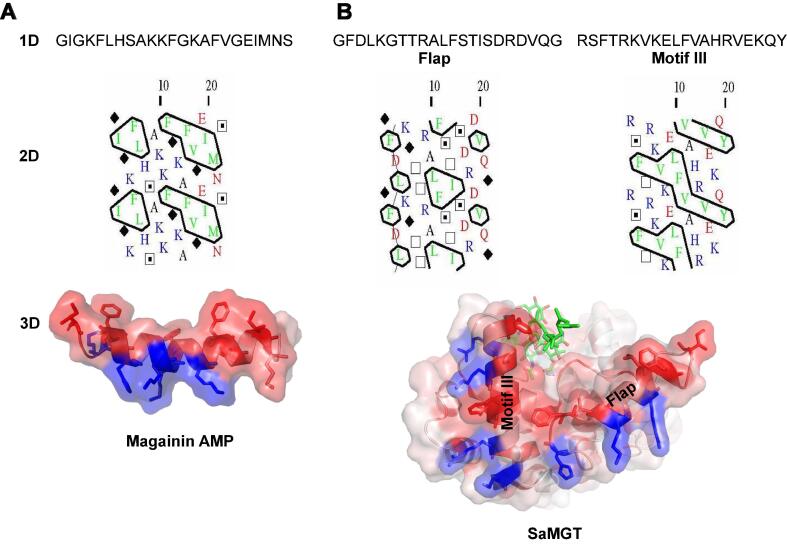
Comparison of the structural and physicochemical features between (A) the cationic α-helical AMP Magainin and (B) the membrane-interacting surface (flap and motif III) of the jaw subdomain in PGT. In both a and b, the upper panel (1D) shows the amino acid sequence, the middle panel (2D) shows the hydrophobic cluster analysis plot and the lower panel (3D) shows the 3D structure with surface representation and amino acid (hydrophobic coloured red and positively charged coloured blue) side chains shown as sticks. (For interpretation of the references to colour in this figure caption, the reader is referred to the web version of this article.)

Clusters C3 and C5 belong to the flap segment and motif III respectively and form the base of the jaw subdomain that makes direct contacts with the membrane-anchored lipid II substrate. A notable feature of these two clusters is the presence of charged and polar residues in the vicinity of the hydrophobic residue clusters. Additionally, the shapes of the hydrophobic clusters C3 and C5 indicate a strong preference for α-helical structures, which agrees with the 3D structures of the PGT domain. Taken together, this suggests that the flap and motif III α-helices have amphipathic properties (Fig. 5B), making the membrane-interacting surface of PGTs amphiphilic for proper adsorption onto the membrane surface to access lipid II substrate.

### 3.5 Similarity in the physicochemical properties of the membrane-interacting surface of the PGT jaw subdomain and amphipathic cationic α-helical AMPs

The structural similarities between the PP-binding motifs in the flap of SaMGT and AMP nisin prompted us to analyse and compare the physicochemical properties of the membrane-interacting surface of the jaw subdomain with the amphipathic cationic α-helical (ACAH) AMPs in the Magainin family – the largest known group of ACAH AMPs (Fig. 5A). The bacteria-killing activity of ACAH AMPs by segregation of anionic lipids in bacterial membranes through a “charge cluster” mechanism depends on two physicochemical properties: an overall cationic nature for selective recognition of negatively charged target moieties on the cytoplasmic membrane and hydrophobicity for interaction with the fatty acyl chains of phospholipids (Nguyen et al., 2011, Epand et al., 2010).

A 2D HCA of Magainin revealed a distribution pattern of hydrophobic and positively charged residues describing the conserved structural and physicochemical properties of ACAH AMPs (Fig. 5A). Remarkably, we found a strikingly similar 2D pattern by assembling the HCA plots of the flap and motif III of the PGT jaw subdomain close to each other (Fig. 5B). This minimal 2D pattern of the flap and motif III can be found in the HCA plots of the PGT domain in MGTs and class A PBPs (Fig. 4 and Supplementary Fig. 3), suggesting that this possibly could be a minimal structural motif and a physicochemical feature for phospholipid recognition and the neighboring residues in the flap and motif III would help in attaining selectivity or specificity for lipid II substrate. Unfortunately, we could not perform a similar analysis on nisin family due to the presence of lanthionine bridges and several post-translationally modified amino acids, which make it difficult to characterize their physicochemical properties and for 2D HCA analysis.

### 3.6 Molecular dynamics analysis of the PGT domain

#### 3.6.1 The flap region is highly dynamic in apo and MoeA-bound simulations

The flexibility of the full-length SaMGT within the membrane was analyzed by measuring the root-mean-square fluctuation (RMSF) of the α-carbons throughout the 500 ns simulations. Due to the persistent interactions with phosphate groups within the membrane, most of the jaw subdomain was relatively rigid compared to the more solvent-exposed head subdomain (Supplementary Fig. 4).

**Fig. 6:**
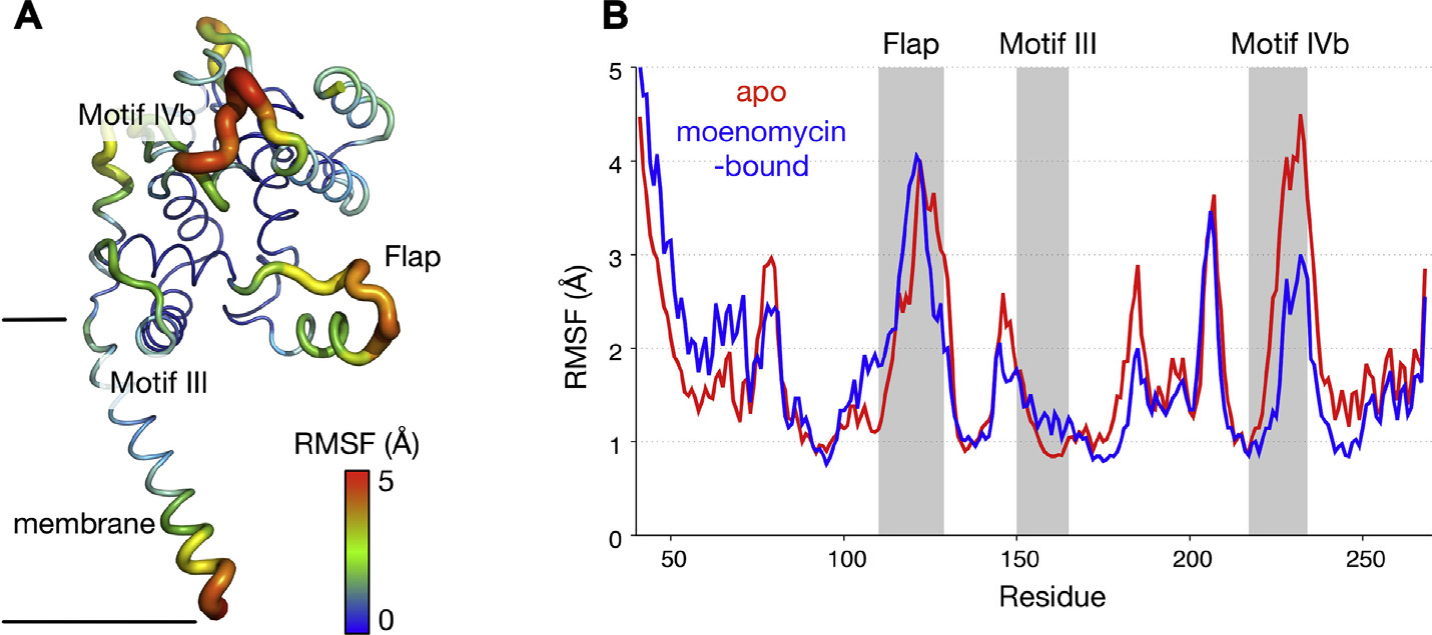
RMSF of SaMGT during MD simulations. (A) Average RMSF values of the α-carbons of apo SaMGT from three 500 ns simulations at 310 K mapped on its structure in ribbon representation. (B) This RMSF values compared to the ones from ${\text{MoeA}}_{\mathrm{DON}}$-bound simulations, with the flap, motif III, and motif IVb highlighted.

Part of the flap region (residues Phe120 to Gln129), however, was significantly more flexible than the rest of the jaw subdomain, with the RMSF values reaching 4 Å (Fig. 6). These residues were exposed to the bulk solution and did not contact the membrane unlike the rest of the flap and motif III. With the ${\text{MoeA}}_{\mathrm{DON}}$ bound in the active site cleft, these residues in flap remained flexible as no significant interactions were formed between them. Another part of the protein that was highly mobile was motif IVb (residues Ala225 to Ser235) in the head subdomain. Unlike the flap, however, these residues were stabilised by the binding of ${\text{MoeA}}_{\mathrm{DON}}$, suggesting that motif IVb residues are important for the MoeA binding to the donor site of the SaMGT catalytic cleft.

#### 3.6.2 The flap region flips towards motif III in extended simulations

**Fig. 7:**
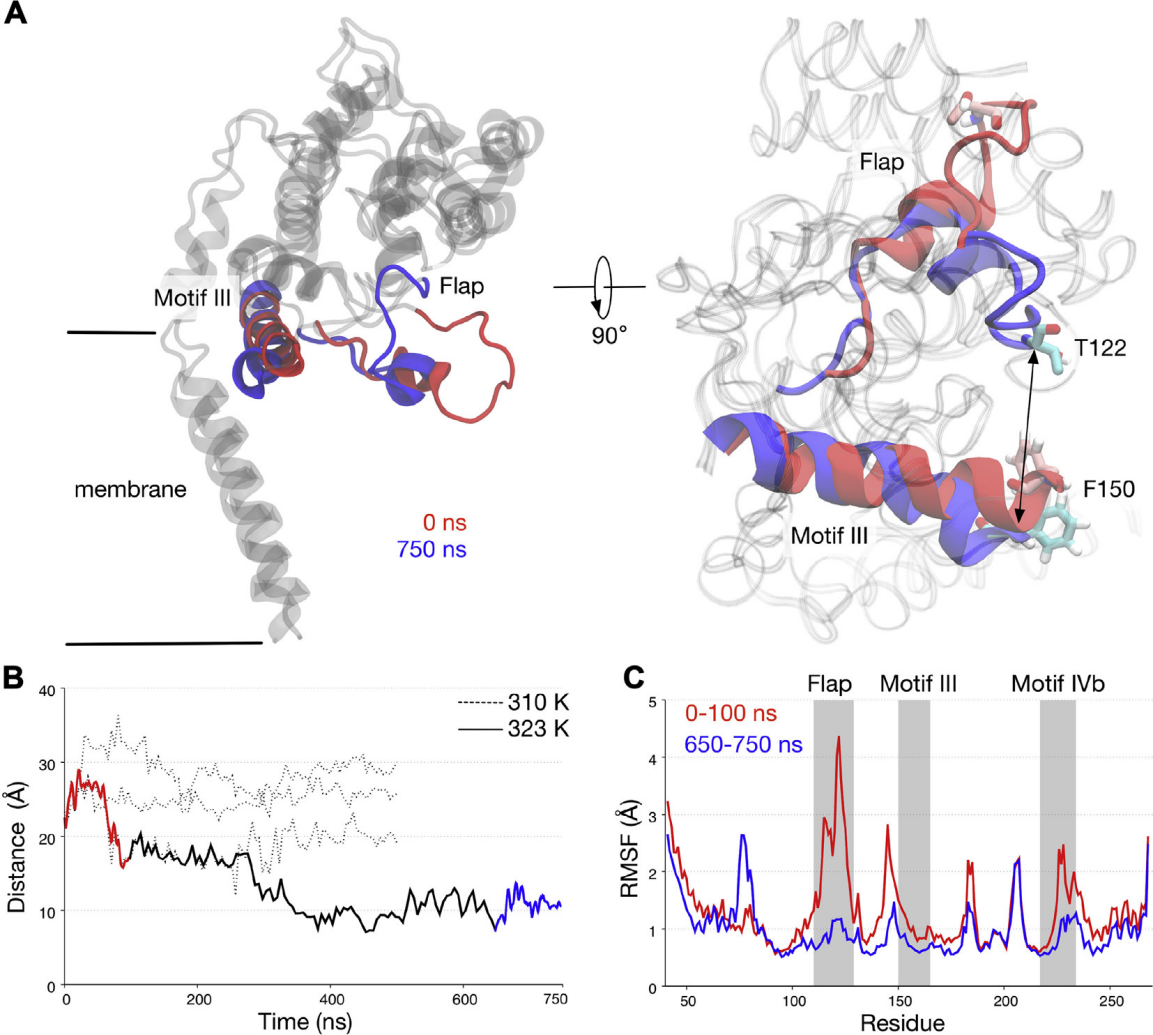
The motion of the flap towards motif III in apo simulation. (A) Snapshots of SaMGT at the beginning and at the end of a 750 ns simulation at 323 K from the plane of the inner membrane (left) and from the extracellular side (right). Two residues found in the flap and motif III (T122 and F150, respectively) are shown in licorice representation and colored pink (0 ns) or cyan (750 ns). (B) The minimum distance between T122 and F150 during three 500 ns simulations at 310 K (dotted line), and one extended 750 ns simulation at 323 K (solid line), as indicated by the arrow in (A). (C) α-carbon RMSF values at the beginning (red) and at the end (blue) of the 750 ns simulation with the positions of the flap, motif III, and motif IVb highlighted. (For interpretation of the references to colour in this figure caption, the reader is referred to the web version of this article.)

The flap region, in particular the soluble and structurally disordered part (residues Phe120 to Gln129), moved in a stochastic manner, i.e. the flap either flipped towards or away from motif III. In one of the 500 ns simulations, we found a significant motion towards motif III, such that some of its residues shifted as far as 10 Å from the crystal structure (Fig. 7). We explored this further by performing one simulation at a higher temperature of 323 K to accelerate the dynamics of the system, and for a longer time scale of 750 ns. This simulation showed an even larger motion of the flap, whereby the flexible part moved around 15–20 Å from its original position towards motif III. Comparing the RMSF values at the beginning and at the end of the simulation indicated that this flipping motion stabilised the flexible flap region, whereby at the end of the simulation the RMSF was reduced from 4 Å to 1 Å. The distance between two reference residues (Thr122 on the flap and Phe150 on motif III) remained around 10 Å during the last 350 ns of the simulation, further corroborating the stabilising effect of this motion towards the flap region. It is possible that under a biological condition, this large conformational change occurs at a longer time scale, perhaps microsecond, which explains why it is only observed in one of the original simulations and requires a higher temperature to further induce this motion. Overall in the apo configuration, the jaw subdomain prefers to be in a resting state, whereby the flap and motif III are in close proximity to each other.

#### 3.6.3 MoeA binding in the donor site was stable throughout the simulations

**Fig. 8:**
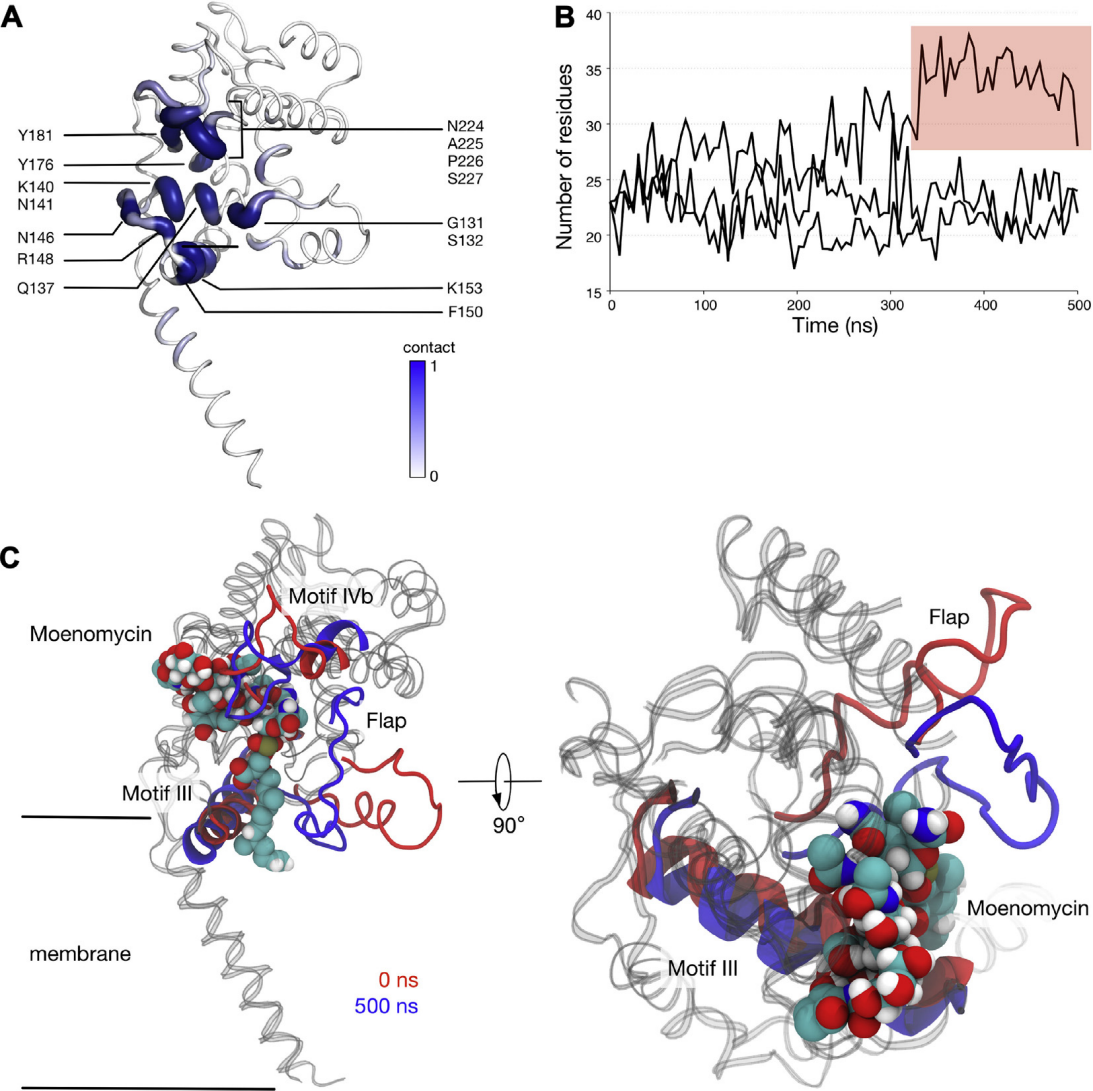
The binding of ${\text{MoeA}}_{\mathrm{DON}}$ to SaMGT. (A) Residues that were in contact with ${\text{MoeA}}_{\mathrm{DON}}$ averaged over three 500 ns simulations. A score of 1 indicates residues that interacted with ${\text{MoeA}}_{\mathrm{DON}}$ throughout the entire simulation, and 0 indicates no contact. Residues that scored 0.8 and above are labelled. (B) The number of residues that bind to ${\text{MoeA}}_{\mathrm{DON}}$ during the three simulations. Highlighted in red is the portion of one simulation whereby the flap interacted with ${\text{MoeA}}_{\mathrm{DON}}$. A distance cut-off of 4 Å was used during these contact analyses. (C) Snapshots of ${\text{MoeA}}_{\mathrm{DON}}$-bound SaMGT at the beginning and at the end of one of the simulations. MoeA is shown in van der Waals representation.

${\text{MoeA}}_{\mathrm{DON}}$ remained bound to the SaMGT catalytic cleft throughout all three 500 ns simulations, whereby most of the interactions were via residues in motif IVb (residues Asn224-Ser227), Lys140 and Asn141 (Fig. 8). The polyprenyl chain of ${\text{MoeA}}_{\mathrm{DON}}$ was stabilised by hydrophobic interactions to Phe150 in motif III, the phosphate group formed salt bridges with Lys140 and Arg148, whilst the sugar moieties bound to several polar residues like Asn141, Tyr176 and Tyr181. Looking at the number of residues in contact with ${\text{MoeA}}_{\mathrm{DON}}$ throughout the simulations, we found only a small degree of fluctuation in two of these simulations, indicating a strong and stable binding. Interestingly in one of them, the number of residues in contact with ${\text{MoeA}}_{\mathrm{DON}}$ increased significantly towards the end of the simulation. Upon further inspection, we found the flap moved around 10 Å towards ${\text{MoeA}}_{\mathrm{DON}}$ resulting in more residues in contact with the ligand. This behaviour is similar to the apo simulation, although the presence of ${\text{MoeA}}_{\mathrm{DON}}$ between the flap and motif III prevented these two regions from approaching each other to the same degree. As such, in the presence of ${\text{MoeA}}_{\mathrm{DON}}$, the jaw subdomain remains open and the flap is likely to be flexible due to the lack of interactions with motif III.

## 4 Discussion

### 4.1 Recognition and binding of lipid II by PGT enzymes

The schematic models describing PGT processive catalytic mechanism for glycan strand synthesis (Yuan et al., 2007, Huang et al., 2012, King et al., 2017) generally consider that, to start the catalytic cycle, the donor and the acceptor sites will be occupied by the respective lipid II molecules. However, lipid II is a low-abundant molecule in comparison with the different types of phospholipids present in several fold access in the bacterial cell membrane. Thus, none of the proposed schematic models provide an obvious explanation for how the PGT enzymes access the membrane-bound lipid II and selectively capture them into the active site. Crystal structures of several PGTs (Lovering et al., 2007, Yuan et al., 2007, Lovering et al., 2008, Yuan et al., 2008, Heaslet et al., 2009, Sung et al., 2009, Fuse et al., 2010, Han et al., 2011, Huang et al., 2012, King et al., 2017) suggest that the motifs I, II, III and flap region of the jaw subdomain interact with the cytoplasmic membrane in a region distinct from the transmembrane α-helix and are required for the recognition of lipid II. *In vitro* experiments by Thierry Vernet and co-workers on the PGT domain of *Streptococcus pneumoniae* PBP1b (Di Guilmi et al., 2003) and PBP2a (Di Guilmi et al., 2003) showed that motif I stabilizes the PGT domain and together with motif II play a crucial role in the efficient recognition and binding of lipid II. In our re-refined model (PDB ID code  6FTB), residues Asp101 (motif I) and Gln136 (motif II) make hydrogen bond interactions with ${\text{MoeA}}_{\mathrm{JAW}}$ and mutating these residues Asp101Asn and Gln136Glu abolished lipid II polymerization activity (Heaslet et al., 2009). Our HCA analysis of the PGT domain in MGTs and class A PBPs (Fig. 4 and Supplementary Fig. 3) revealed that placing the flap and motif III in close proximity produces a 2D pattern of hydrophobic and positively charged residues describing a conserved structural and physicochemical properties similar to ACAH AMPs (Fig. 5). This possibly could be a minimal structural motif and a physicochemical feature of the jaw subdomain for phospholipid recognition and that the other neighboring residues in the flap and motif III may help in attaining selectivity or specificity for lipid II. Further, the stochastic movement of the flap closer to or away from motif III in our MD simulations is suggestive of a charge cluster-like mechanism by the jaw subdomain to segregate lipid II substrate from the crowded milieu of other phospholipids in the bacterial membrane (Fig. 7, Fig. 8).

Most AMPs are thought to be linear and unstructured in aqueous solution and adopt a stable amphipathic conformation in the membranous environment for making target substrate specific interactions (Nguyen et al., 2011). This dynamic behavior (folding and unfolding property) of AMPs is necessary for the fine-tuning of structural parameters (peptide conformation, hydrophobicity, hydrophobic moment, peptide charge and the size of the hydrophobic/hydrophilic domain) to attain substrate selectivity and specificity. Most of the crystal structures of PGT in apo form or in complex with MoeA show an unstructured flap and weak electron density for the motif III α-helix, indicating the dynamic nature of the jaw subdomain at the membrane-water interface. This suggests that, like AMPs, the flap and motif III seem to require a membranous environment to maintain the secondary structure and stability for efficient lipid II binding and elongation mechanism. MD analysis showed that the jaw subdomain was relatively rigid through stable interactions with phosphate groups embedded in the membrane (Supplementary Fig. 4). Likewise, the presence of dimethyl sulfoxide (DMSO), which is a good membrane-mimicking solvent (Duarte et al., 2008) for increased solubility of lipid II substrate and to facilitate faster exchange between detergent micelles, showed a strong influence on the *in vitro* PGT assay (Schwartz et al., 2002, Helassa et al., 2012).

Our discovery of ${\text{MoeA}}_{\mathrm{JAW}}$ bound to the SaMGT jaw subdomain by X-ray crystallography (Fig. 1B) strongly suggests a re-evaluation of the hypothesis proposed by Heaslet et al. that a lipid II could bind in the pocket where phosphate ion was modeled in the PDB ID code  3HZS (Heaslet et al., 2009). Hydrogen bond interactions between the phosphate ion of ${\text{MoeA}}_{\mathrm{JAW}}$ and the backbone atoms of the well-conserved motif I residues implies the presence of a common PP-binding structural motif in all PGT enzymes for lipid II recognition and binding (Supplementary Fig. 1). In the re-refined SaMGT model, the ring A and B of ${\text{MoeA}}_{\mathrm{JAW}}$ occupies the same pocket as the Mg^2+^ ion in the structure of SaMGT bound with lipid II analog in the acceptor site (PDB ID code  3VMT, chain A) (Huang et al., 2012) suggesting a significant functional role(s) for this pocket made by the motifs I, II and III. The motif I PP-binding site in the PGT jaw subdomain is structurally similar to the “PP cage” motif of nisin. Nisin binds to lipid II by docking onto the PP moiety (Bauer and Dicks, 2005, Breukink and de Kruijff, 2006) because the PP group remains stably anchored at the lipid-water interface in the same plane as the phosphate groups of the other membrane lipids, whereas the lipid II polar head-group (GlcNAc-MurNAc) and the hydrophobic bactoprenol tail are dynamic and display a high degree of conformational heterogeneity (Chugunov et al., 2013, Witzke et al., 2016). This suggests that the PGT jaw subdomain would use the motif I PP-binding site for docking onto the PP moiety of the substrate (lipid II or lipid IV) to properly anchor onto the membrane surface. The docking may help the PGT domain to achieve a slight stiffening to control the global dynamic motions, which in turn would allow the PGTs to position their active site at the correct height from the membrane surface to interact with the polar head-group of the lipid II substrate. MD analysis showed that the residues in the “tip” of the flap made contacts with the phosphate groups of the lipids embedded in the membrane (Supplementary Fig. 4), which further corroborates that the flap segment can partially insert into the membrane bilayer to access lipid II. Such a mode of binding of the PP moiety would allow the bactoprenol tail of the lipid II to stabilize against the continuous hydrophobic surface (residues Phe110, Leu112, Leu119 and Phe120 in SaMGT) along the axis of the flap α-helix, similar to the acyl tail of the detergent lauryldimethylamine *N*-oxide (LDAO) stabilized by hydrophobic interactions from residues in the SaPBP2 flap α-helix (Fig. 3B) (Lovering et al., 2008). The backbone amide atoms of Phe110 and Leu112 are essential for lipid II binding (Heaslet et al., 2009) and a double mutation Leu119Asn-Phe120Ser in SaMGT abrogates its interaction with lipid II and MoeA and a complete loss of specific MoeA binding at the donor site (Bury et al., 2015).

### 4.2 Positive cooperativity and allostery in the PGT active site

**Fig. 9:**
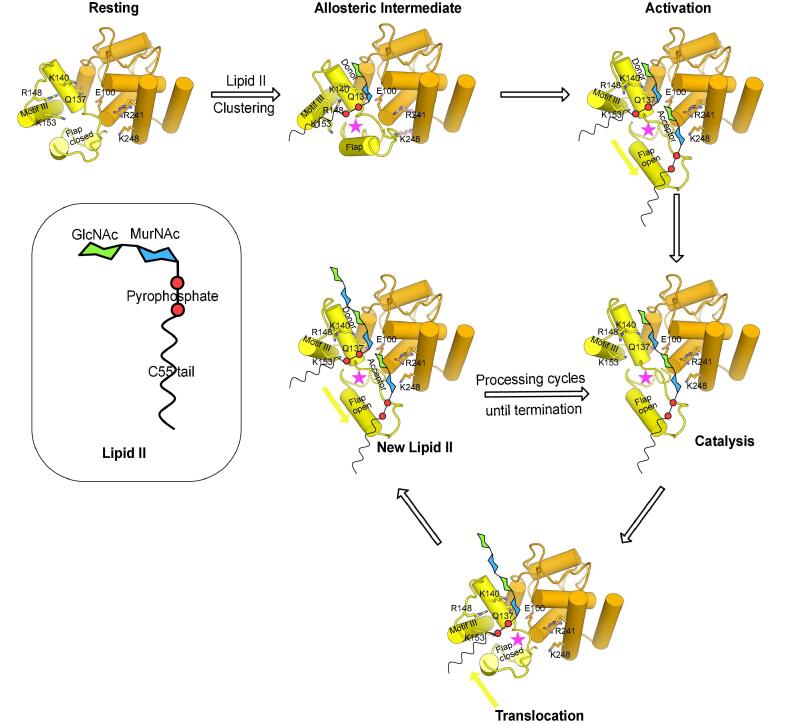
Hypothetical model illustrating the processive mechanism of lipid II polymerization by PGT. Structure of lipid II is shown in the inset. Cartoon representation of PGT (α-helices shown as cylinders) showing the head subdomain (colored orange) and the jaw subdomain (colored yellow). The key active site residues are shown as sticks and coloured by atom type. The flap and motif III helices are labelled. The putative allosteric site is shown as a star (colored pink). The movement of the flap to show open and closed orientation is indicated by arrows (colored yellow). PGT conformer representing the respective functional state approximate the select experimentally determined PGT structures and are annotated as follows: Resting and Translocation (PDB ID code 3DWK chains B and C), Allosteric Intermediate (PDB ID codes 3VMQ chain A, 2OQO), Activation, Catalysis and New Lipid II (PDB ID codes 3HZS, 3VMT chain A). (For interpretation of the references to colour in this figure caption, the reader is referred to the web version of this article.)

The interaction of MoeA and other potential PGT inhibitors to full-length and truncated variants of class A PBPs and MGT has been extensively studied using various techniques: nuclear magnetic resonance (Rühl et al., 2003, Mesleh et al., 2016), surface plasmon resonance (SPR) (Stembera et al., 2002, Cheng et al., 2008, Bury et al., 2015), fluorescence anisotropy (Cheng et al., 2008) and ITC (Huang et al., 2012). Studies have shown that the PGT transmembrane α-helix contributes to the MoeA binding in the PGT active site (Cheng et al., 2008, Huang et al., 2012). SPR data analysis of MoeA binding to immobilised full-length EcPBP1b by fitting to a 1:1 interaction model resulted in a ${\text{K}}_{d}$ of 0.44 μM (Cheng et al., 2008). Interestingly, the SPR data analysis of full-length SaMGT binding to immobilised MoeA showed a clear inhibition of SaMGT binding by MoeA at 0.5 μM concentration, but the binding curves could not be reconciled by a 1:1 interaction model (Bury et al., 2015). The ITC experiment by Che Ma and co-workers showed a ${\text{K}}_{d}$ of 0.2 μM for MoeA binding to full-length SaMGT, but the authors did not report a binding stoichiometry (Huang et al., 2012). To resolve this apparent discrepancy in binding stoichiometry and to verify the observation of the second MoeA in our re-refined structure (PDB ID code  6FTB), we performed ITC of MoeA binding to full-length SaMGT. Our ITC data has shown two MoeA binding events with positive cooperativity in the SaMGT active site (Fig. 2). Further, in our ITC data analyses, the MoeA binding isotherm could only be fitted using a sequential binding site model (Fig. 2) and the apparent ${\text{K}}_{d}$ of 0.54 μM observed for the ${\text{MoeA}}_{\mathrm{DON}}$ binding event. Based on the SPR results, Bury et al. proposed that lipid II analogs, at low concentrations, preferentially bind to the acceptor site and stabilize the flap region resulting in a structural rearrangement to increase the affinity of the donor site to MoeA (supposed to mimic the growing glycan chain/ lipid IV). This suggested a positive cooperativity between the acceptor and the donor sites and an allosteric activation of donor site in PGTs (Bury et al., 2015). In contrast, our MD analysis of the apo SaMGT model show that in the resting state, the flap is flexible and prefers to be positioned closer to motif III (Fig. 7, Fig. 9, and PDB ID code  3DWK chains B and C) and in this conformation the acceptor site is not available for lipid II binding. *In vitro* experiments using the synthetic lipid II and lipid IV derivatives have shown that the formation of first lipid IV is the rate-limiting step in PG synthesis, probably because lipid II is not an optimal substrate in the donor site for the initiation step and that the rate-limiting step can be changed using longer glycan strands such as lipid IV (Wang et al., 2011). Moreover, lipid IV in isolation has been shown to be a substrate for PGT enzymes (Wang et al., 2011, Barrett et al., 2007). This suggested that binding of lipid IV in the donor site induces structural rearrangements, including probably in the acceptor site that enhances the binding of lipid II during the elongation phase of processive glycan chain polymerization (Sauvage and Terrak, 2016). MD analysis of the apo SaMGT model suggests that the dynamic movements of the motif IVb (Fig. 6) would prevent lipid II from binding to the donor site and this could be the possible reason behind the rate-limiting step during the initiation step. SaMGT pre-incubated with Gal-lipid IV enhanced the lipid II polymerization reaction rate by >6-fold as measured by an *in vitro* PGT polymerisation assay (Wang et al., 2011). Interestingly, in our ITC experiment measuring binding affinities, the ${\text{MoeA}}_{\mathrm{JAW}}$ binding enhanced the ${\text{MoeA}}_{\mathrm{DON}}$ binding by ≈20-fold (Fig. 2). Taken together, we propose that our newly discovered ${\text{MoeA}}_{\mathrm{JAW}}$-binding site, upon binding either lipid II or lipid IV, may induce allosteric motions to reorganize the GT active site and maintain the positive cooperativity between the donor and acceptor sites throughout the lipid II polymerization process. This would result in increase in the affinity of the donor and acceptor sites for lipid II in the initiation step and the substrate binding would shift the dynamic equilibrium by preferentially stabilising a particular motion between the head and jaw subdomains for processive glycan chain elongation. Additionally, we believe that our re-refined SaMGT-MoeA model (PDB ID code  6FTB), specially the ${\text{MoeA}}_{\mathrm{JAW}}$ bound in the jaw subdomain, may help in the structure-based design and development of novel PGT inhibitors.

### 4.3 Mechanistic hypothesis for processive lipid II polymerization by PGT

Based on the above observations (including the amino acid mutations within the active site and jaw subdomain (Heaslet et al., 2009, Bury et al., 2015)), we now propose a mechanistic hypothesis for the processive lipid II polymerization by PGTs (Fig. 9). Our MD data indicate that in the substrate lipid II unbound state (resting state), the flap and motif III lie in close proximity to each other. This, we speculate, would enable the jaw subdomain to build a high local density of positive charge for anionic lipid II accumulation closer to the PGT active site. PGT jaw subdomain docking onto the PP moiety of the membrane-embedded lipid II/lipid IV would be the primary driving force behind – (i) the global structural rearrangement of the PGT active site for increased lipid II affinity (activation state) and (ii) the coupling of the head- and the jaw subdomain concerted motion to regulate the movements of the conserved motifs I–V and allosteric switch between the open and closed conformations of the flap throughout the lipid II polymerisation cycles until termination. A key motion for the processive function would be the swaying action of the flap region towards (closed) and away (open) from the motif III (Fig. 9). Flap opening would carry along with it, a lipid II molecule at the glycosyl acceptor site and allowing another lipid II molecule to bind into the glycosyl donor site, in quick succession, resulting in a pre-catalytic complex. The departure of the C55-PP leaving group from the donor site upon formation of a new β-1,4-linked GlcNAc-MurNAc (catalysis state) followed by the flap closing motion, presumably to attain the resting state, would result in the translocation of the polymerized glycan strand from the acceptor site to the donor site. In one of our MD simulation studies, the flap moved closer to ${\text{MoeA}}_{\mathrm{DON}}$ (supposed lipid IV analog) bound in the donor site (Fig. 8B and C), further corroborating this motion. The occupancy of the donor site would signal the flap to switch to an activation state, carrying a new lipid II molecule at the glycosyl acceptor site for another cycle of transglycosylation reaction thereby facilitating the processive glycan chain synthesis. In conclusion, we propose that a concerted structured and unstructured movement between the head subdomain (motif IVb) and the jaw subdomain (flap) would be essential to accommodate the elongating glycan strand in the active site cleft during the translocation step and for pushing the elongating glycan strand out of the exit channel for subsequent transpeptidase crosslinking reactions by class A and/or class B PBPs.

## Funding

This work was supported by the BBSRC grants BB/M029573/1 (to S.K.) and BB/N003241/1 (to D.I.R.). The computing in this project made use of time on ARCHER granted via the UK High-End Computing Consortium for Biomolecular Simulation, HECBioSim ( http://hecbiosim.ac.uk), supported by EPSRC (Grant No. EP/L000253/1).

## Author contributions

A.S.P. and D.I.R. designed research; A.S.P. and F.S. performed research; A.S.P., F.S., A.J.L., C.G.D., D.J.S., S.K. and D.I.R. analyzed data; and A.S.P., F.S., S.K. and D.I.R. wrote the manuscript.

## Data reference

PDB:  6FTB

## Conflict of interest

None.
