# Supplementary material for: The role of the jaw subdomain of peptidoglycan glycosyltransferases for lipid II polymerization
Source: Cell Surf. 2018 Jun 20;2:54–66. doi: 10.1016/j.tcsw.2018.06.002 (PMC6053601; doi:10.1016/j.tcsw.2018.06.002)
Supplement: Supplementary data 2 [file mmc2.docx]

**The role of the jaw subdomain of peptidoglycan glycosyltransferases for lipid II polymerization**

Avinash S. Punekar^a^, Firdaus Samsudin^b^, Adrian J. Lloyd^a^, Christopher G. Dowson^a^, David J. Scott^c,d^, Syma Khalid^b^, and David I. Roper^a^

^a^ School of Life Sciences, University of Warwick, Coventry CV4 7AL, United Kingdom; ^b^ School of Chemistry, University of Southampton, Southampton SO17 1BJ, United Kingdom; ^c^School of Biosciences, University of Nottingham, Sutton Bonington Campus, Leicestershire LE12 5RD, United Kingdom; ^d^ISIS Neutron and Muon Spallation Source and Research Complex at Harwell, Rutherford Appleton Laboratory, Oxfordshire, United Kingdom

**SUPPLEMENTARY INFORMATION**


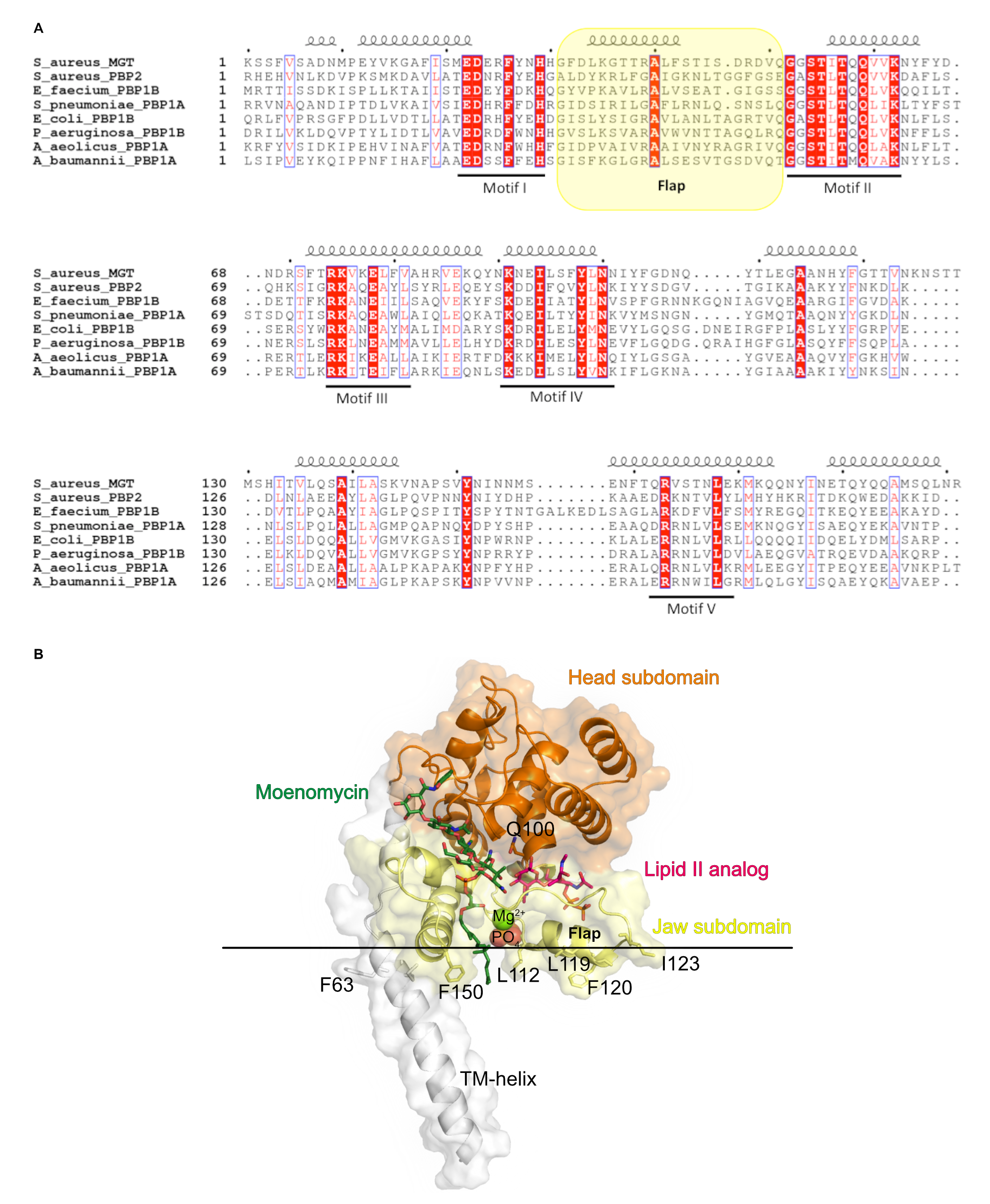


Fig. 1. (A) Multiple sequence alignment of PGT domain in monofunctional glycosyltransferases and class A pencillin-binding proteins. The pale yellow background indicates the protruding dynamic 'flap' region in the jaw subdomain. Conserved residues are shown in white on red background, and conservative substitutions are in red. Secondary structure of *Staphylococcus aureus* monofunctional glycosyltransferase (SaMGT) is indicated above the alignment, and sequence motifs of PGT domain are indicated below the alignment. (B) Overall structure of ligand-bound SaMGT. Model was made by superimposing and merging PDB ID codes 3HZS and 3VMT. The TM-helix is colored gray and the jaw subdomain and the head subdomain are colored pale yellow and orange respectively. MoeA_DON_ (green) in the donor site and lipid II analog (pink) in the acceptor site are shown as stick. Magnesium and phosphate ions are shown as spheres. The residues located at the water-membrane interface are shown as sticks, labeled and colored by atom-type.


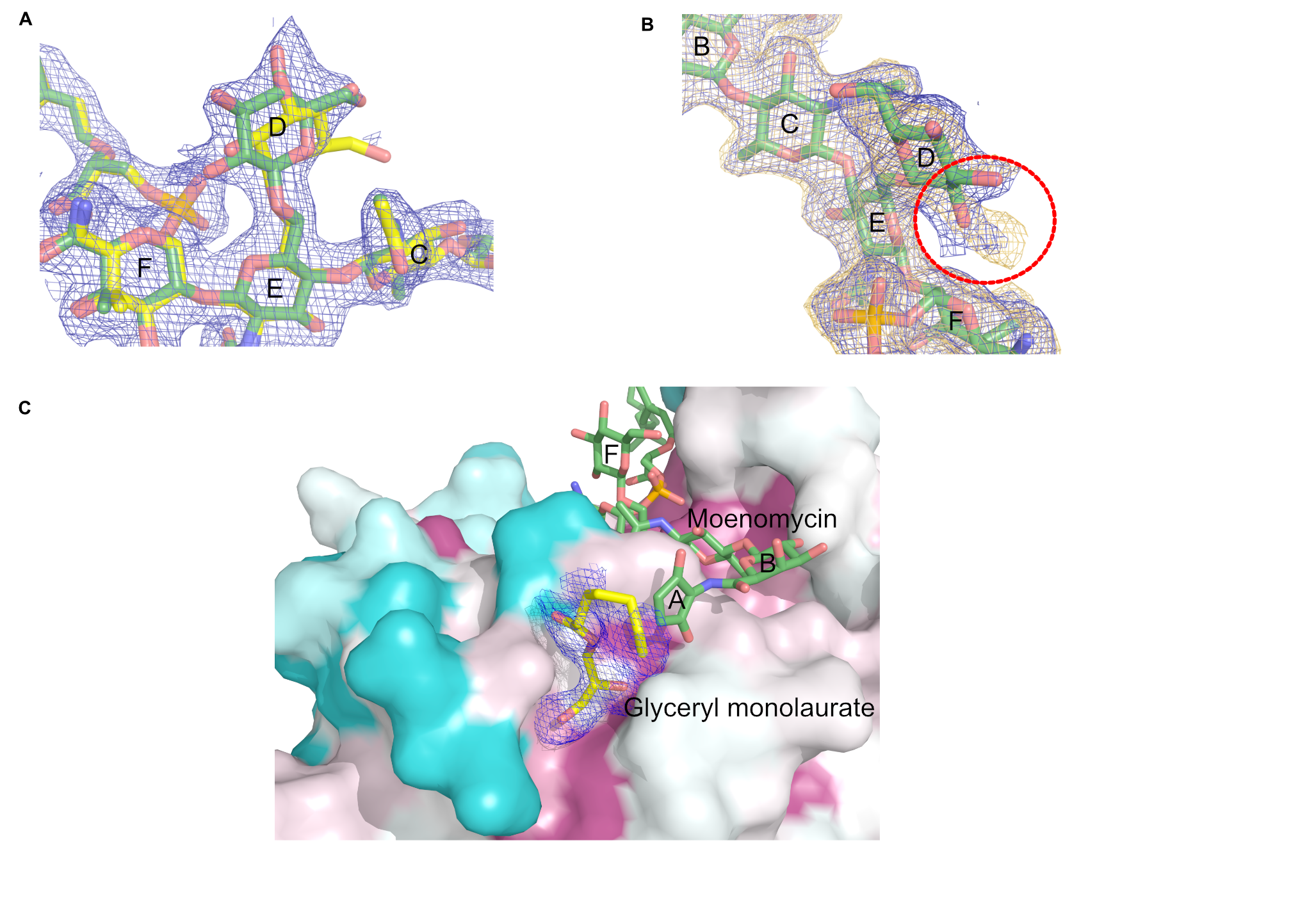


Fig. 2. (A) MoeA_DON_ in PDB ID code 6FTB (colored green atom type) and PDB ID code 3HZS (colored yellow atom type). Marginal shift in the ring D to fit correctly into the 2Fo-Fc electron density map (blue mesh) contoured at 1σ. For clarity, SaMGT is not shown. (B) Polder map (orange mesh) for MoeA_DON_ is contoured at 2σ and the 2Fo-Fc map (blue mesh) is contoured at 1σ. The bifurcated electron density is marked by a red dotted circle. (C) The 2Fo-Fc map (blue mesh) for glyceryl monolaurate (colored yellow atom type) is contoured at 1σ. MoeA_DON_ (colored green atom type) is bound in the donor site. The surface of SaMGT is colored according to sequence conservation using ConSurf. The color spectrum ranges from magenta (highest conservation) to cyan (lowest conservation).


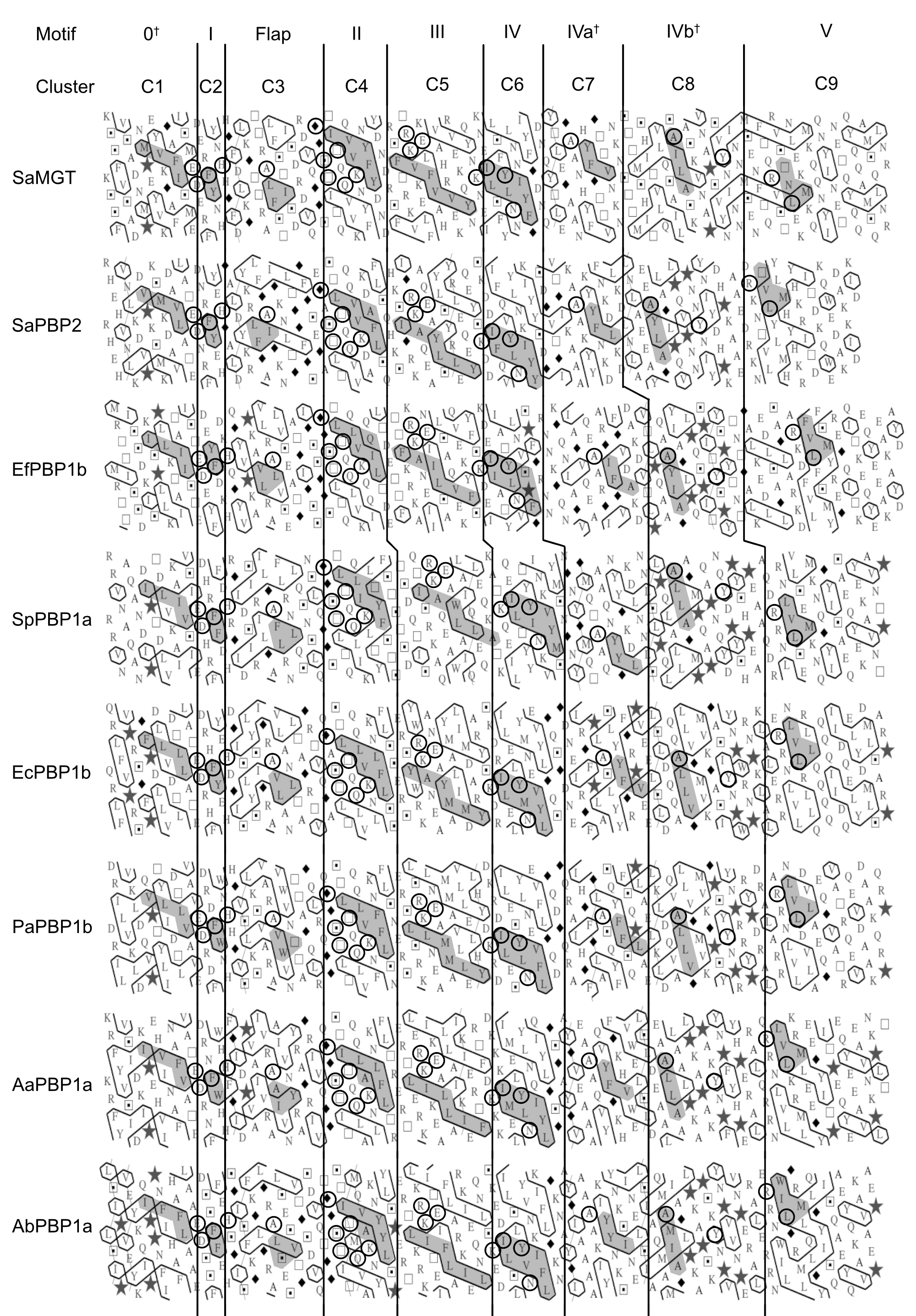


Fig. 3. Aligned HCA plots of the PGT domain in monofunctional glycosyltransferases (MGTs) and class A penicillin-binding proteins (PBPs). The standard one-letter code for amino acids is used except for glycine (◆), proline (★), serine (⚀) and threonine (☐) respectively. The vertical lines indicate proposed correspondence between the compared sequences. The strictly conserved residues (in the multiple sequence alignment Fig. 1a) are indicated with a black circle. The conserved and conservatively substituted amino acids within the hydrophobic clusters (shaded in gray) suggest a structural and functional relationship. † indicates motifs identified in this study. UniProt accession numbers: *Staphylococcus aureus* MGT (SaMGT, Q7A0I6), *S. aureus* PBP2 (SaPBP2, Q9R744), *Enterococcus faecalis* PBP1b (EfPBP1b, I3U3N7), *Streptococcus pneumoniae* PBP1a (SpPBP1a, Q9WW11), *Escherichia coli* PBP1b (EcPBP1b, P02919), *Pseudomonas aeruginosa* PBP1b (PaPBP1b, A6VCF4), *Aquifex aeolicus* PBP1a (AaPBP1a, O66874) and *Acinetobacter baumannii* AbPBP1a (AbPBP1a, G1C794).


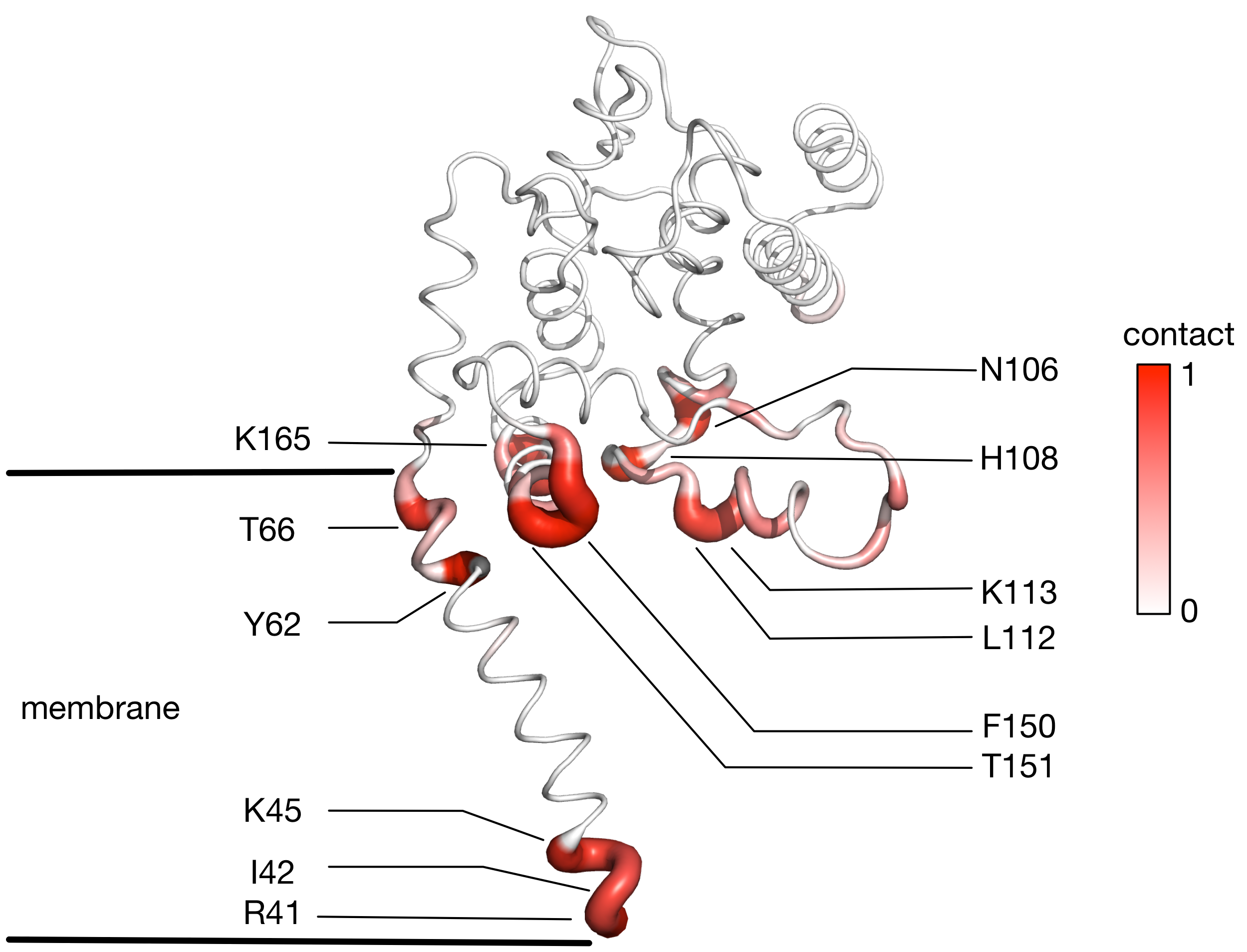


Fig. 4: Average contacts made between SaMGT and the phosphate groups of the lipids inside the membrane, with 1 indicating contacts made throughout the 500 ns simulation, and 0 indicating no contact. Distance cut-off used for this contact analysis was 4 Å. The approximate position of the membrane is indicated by the two thick lines.

Table 1. Refinement statistics

| Structure | 3HZS* | 6FTB |
| --- | --- | --- |
| *Refinement* |  |  |
| Resolution range (Å) | 23.12 - 2.1 | 23.12 - 2.1 |
| No of reflections | 18,121 | 19,082 |
| % reflections (test set) | 5.0 | 5.0 |
|  |  |  |
| *Number of atoms* |  |  |
| Protein | 1708 | 1717 |
| Water | 124 | 141 |
| Other | 105 | 206 |
| R_work_ (%) | 19.0 | 19.85 |
| R_free_ (%) | 24.3 | 24.03 |
|  |  |  |
| *Average B-factor (Å^2^)* |  |  |
| Protein | 35.9 | 38.2 |
| Water | 40.3 | 49.4 |
| Other | 37.7 | 63.5 |
|  |  |  |
| RMSD from ideal bond length (Å) | 0.019 | 0.010 |
| RMSD from ideal bond angle (deg) | 1.80 | 1.05 |
| *Ramachandran plot* |  |  |
| Favored(%) | 96.46 | 98.1 |
| Allowed(%) | 3.54 | 1.9 |
| Outliers(%) | 0 | 0 |
|  |  |  |

* Refinement statistics are reported in (8)

Table 2: List of simulations performed

| System | Simulation time (ns) | Temperature (K) |
| --- | --- | --- |
| *apo* | 3 X 500 | 310 |
| *apo* | 1 X 750 | 323 |
| Moenomycin-bound | 3 X 500 | 310 |

**Cloning, Expression and Purification of SaMGT**

SaMGT (residues Q28-R269) was cloned into the pET15b (EMD Biosciences) vector as described previously (12) and the expression vector was transformed into *E. coli* BL21 Star^TM^ (DE3) cells. An overnight pre-culture grown in LB broth medium (supplemented with 100 μg/mL ampicillin) was used to inoculate 2YT broth auto-induction media (Formedium, UK) supplemented with antibiotic and the culture was grown at 37°C. When OD_600_ reached 1.0, the temperature was lowered to 18°C and the incubation was continued overnight (≈ 18 h). All purification steps were done at room temperature. The cell pellet was resuspended in the lysis buffer (20 mM Tris-HCl, pH 8.0, 200 mM NaCl, 2 mM phenylmethylsulfonyl fluoride (PMSF), 15 mM imidazole and 40 mM 3-[(3-Cholamidopropyl)dimethylammonio]-1-propanesulfonate (CHAPS), lysed by sonication and the cell debris was cleared by centrifugation. Supernatant was filtered through 0.2 μm filter and loaded onto HisTrap HP 5 mL column (GE Healthcare). The column was washed with 100 mL buffer A (20 mM Tris-HCl, pH 8.0, 200 mM NaCl, 2 mM N-decyl-β-d-maltopyranoside (DM)) containing 120 mM imidazole and SaMGT was eluted with 50 mL of buffer A containing 500 mM imidazole. Purified SaMGT was dialysed against buffer A overnight at 4°C to remove imidazole. SaMGT was concentrated using Thermo Scientific^TM^ Pierce^TM^ ultrafiltration centrifugal protein concentrators (molecular weight cutoff 10000) and the N-terminal 6xHis-tag was removed by thrombin cleavage. SaMGT was further purified using Superdex 75 Increase 10/300 GL column pre-equilibrated with buffer A.
